# Association between intergenerational solidarity involving elders and mental health of Indigenous people living off reserve

**DOI:** 10.1186/s12889-022-12887-6

**Published:** 2022-03-16

**Authors:** Chantal Viscogliosi, Hugo Asselin, Lise Trottier, Monia D’Amours, Mélanie Levasseur

**Affiliations:** 1 School of Rehabilitation, Faculty of Medicine and Health Sciences, Université de Sherbrooke, Sherbrooke, Quebec Canada; 2 Research Centre on Aging, Centre intégré universitaire de santé et de services sociaux de l’Estrie-Centre hospitalier universitaire de Sherbrooke, Sherbrooke, Canada; 3 School of Indigenous Studies, Université du Québec en Abitibi-Témiscamingue, Rouyn-Noranda, Canada

**Keywords:** Indigenous health services (MeSH), Intergenerational relations (MeSH), Aged (MeSH), Child health (MeSH)/adolescent health (MeSH), Population health (MeSH), Mental health (MeSH)

## Abstract

**Background:**

Indigenous elders play an important role in transmitting knowledge, values and practices, hence fostering identity-building through intergenerational solidarity. We aimed to verify the association between intergenerational solidarity involving Indigenous elders and mental health of Indigenous people living off reserve.

**Methods:**

We carried secondary analyses of data for a subsample from the cross-sectional 2012 Aboriginal Peoples Survey (total sample: *n* = 28,410 Indigenous persons aged ≥6 years old living off reserve; subsample: *n* = 13,020 aged 18–44 years old). Controlling for age as well as material and social deprivation, we used logistic regressions to verify the association between intergenerational solidarity (proxied as time spent with an elder and potential of turning to an elder or grandparent for support in times of need) and mental health (perceived mental health, mood disorders, anxiety, suicidal thoughts and attempts).

**Results:**

About 39 and 9% of the respondents respectively reported having spent time with an elder and would have turned to an elder or grandparent for support in times of need. Women who would not turn to an elder or grandparent for support in times of need were more likely to report fair or poor perceived mental health (OR = 1.69, *p* = 0.03). Men not spending time with an elder were more likely to experience mood disorders (OR = 1.66, *p* = 0.004). Women who would not turn to an elder or grandparent for support in times of need were more likely to experience anxiety disorders (OR = 1.57, *p* = 0.04). Women not spending time with an elder or who would not turn to an elder or grandparent for support in times of need were respectively more likely to have suicidal thoughts (OR = 1.62, *p* = 0.04) or to have attempted suicide (OR = 3.38, *p* = 0.04).

**Conclusion:**

Intergenerational solidarity is associated with better mental health outcomes of Indigenous people living off reserve. These results could guide policies and practices that aim to enhance mental health and wellness in Indigenous populations.

## Background

Indigenous people generally face more important mental health and wellness issues than the non-Indigenous population in Canada, as a result of disparities in living conditions, education, housing, and economic development [1–4]. These disparities are enhanced by the fact that the theories and practices used in western health services cause inequities, cultural insecurity, and even harm to Indigenous people [5, 6]. Monitoring the determinants of Indigenous health, such as education and relationships with family and friends, is key to developing appropriate health services [7]. Moreover, culturally sound health services need to be implemented, as approaches based on western science fail to properly address the issues experienced by Indigenous people [8, 9]. For this purpose, holistic approaches that consider all dimensions of wellness and focus on the strengths of individuals and communities may produce better results than interventions focusing on specific problems without considering interactions between dimensions [10].

Elders play an important role in Indigenous communities, notably by transmitting knowledge, values, and culture, hence fostering identity-building through intergenerational solidarity [11–14]. The social participation of elders thus contributes to the development of positive attitudes and behaviors in Indigenous communities [13]. More specifically, Indigenous elders contribute to the development of inner strength, resilience, harmony, self esteem, meaning of life, emotion management, family relationships, as well as formal and informal education [13]. Studies with Indigenous and non-Indigenous elders have shown that their involvement in intergenerational activities provides benefits to themselves (functional and intellectual skills), but also to youth (meaning of life), families (development of a network) and communities (vitality, governance) [15–17]. Hence, intergenerational solidarity involving elders should be considered to develop holistic approaches to healthcare and wellness [18, 19]. Intergenerational solidarity is composed of intimacy (affectual solidarity), agreement (consensual solidarity), dependency (functional solidarity), integration (associational solidarity), opportunities for interaction (family solidarity), and familism (normative solidarity) [20]. Intergenerational solidarity is part of more general social support, which is the perception of being part of a network encompassing four functions: emotional, tangible, informational and companionship [21].

Holistic approaches to health and wellness based on the contribution of Indigenous elders to intergenerational solidarity could positively influence mental health of community members, for example, reducing mood disorders, anxiety, and suicide [22, 23]. To benefit from the role that elders can play in the health and wellness of Indigenous people, it is essential to have a better understanding of how intergenerational solidarity influences mental health outcomes of Indigenous community members. To our knowledge, none of the studies documenting intergenerational solidarity has investigated these associations so far. Hence, in the present study, we aimed to verify the association between intergenerational solidarity involving Indigenous elders and mental health outcomes of Indigenous people living off reserve.

### Theoretical framework

From a health promotion perspective, our study is based on the International Classification of Functioning, Disability and Health (ICF) of the World Health Organization (WHO) [24]. The ICF describes the functioning of individuals in their activities and areas of life in interaction with personal factors, health problems, organic functions, and environmental factors. In the ICF model, the functioning of individuals in their activities and areas of life has bi-directional influences with health, and specifically with mental health. Age, gender, and socioeconomic conditions affect activities and mental health outcomes [25, 26]. Hence, we investigated the association between intergenerational relationships (i.e., activities and areas of life) and mental health outcomes of Indigenous men and women living off reserve, adjusting for age and for material and social deprivation.

## Methods

We conducted secondary analyses of data retrieved from the Aboriginal Peoples Survey (APS) conducted in 2012 by Statistics Canada [27]. More than 50,000 individuals were selected to participate in this cross-sectional survey, and data on socioeconomic conditions were obtained from 28,410 Indigenous respondents aged 6 years and older, living off reserve in private dwellings in Canada. The sample design used stratification-specific domains of estimation (geographical regions, education level and Indigenous group, i.e. First Nation, Metis or Inuit) [27].

As Statistics Canada recommends not using mental health data for respondents under 18 years old for reliability considerations, we retained a subsample of respondents between 18 and 44 years old (*n* = 13,020). Moreover, intergenerational solidarity variables (see below) were only available for participants under 45 years old. Respondents were interviewed on the phone or in person in their home for an average of 40 min (up to 1 h maximum). The interviews were conducted in English, French or Inuktitut, directly or, for respondents having a physical or mental disability, with a proxy.

### Outcome variables related to mental health

Among the variables available in the APS database, those documenting mental health were (1) perceived mental health, (2) mood disorders, (3) anxiety, (4) suicidal thoughts (during the last 12 months) and (5) attempted suicide (during the last 12 months). Outcome variables related to mental health were based on the following questions of the APS: (1) In general, would you say your mental health is … (excellent, very good, good, fair, or poor)? (2) Do you have a mood disorder such as depression, bipolar, mania or dysthymia? (3) Do you have an anxiety disorder such as phobia, obsessive-compulsive or panic disorder? (4) Have you seriously thought about suicide or killing yourself in the past 12 months? (5) Have you attempted suicide or tried to kill yourself in the past 12 months? Perceived mental health (excellent, very good, good, fair, poor) was dichotomized (fair and poor vs. other answers).

### Explanatory variables related to intergenerational solidarity

As intergenerational solidarity was not directly measured in the APS, we used two variables as proxies: (1) had spent time with an elder and (2) would turn to an elder or a grandparent for support in times of need. This second proxy combines two different questions of the APS, one about elder support and one about grandparent support in times of need. The first explanatory variable indicated if (yes/no) the respondent had spent time with an elder outside school hours during the current school year. For those not in school (dropouts or graduates), the reference period was the last year of elementary or high school. The second explanatory variable asked if (yes/no) they would turn to an elder or a grandparent for support in times of need.

### Statistical analyses

We used descriptive statistics to examine the respondents’ socio-demographic, mental health, and intergenerational solidarity profiles. Differences between men and women were tested with a t-test (age) or chi-square test (proportions). To verify the association between *lack of* intergenerational solidarity and mental health *issues*, we used logistic regression with an alpha significance level of 0.05. Because they could influence the association between the outcome and explanatory variables, we controlled for the following variables: age, and material and social deprivation. Developed by Pampalon et al. [28] material and social deprivation indexes respectively considered the proportion of persons without a high school diploma, the proportion employed, and average personal income, as well as the proportion of persons living alone, separated, divorced or widowed, and single-parent families. As recommended by Statistics Canada, we weighted all statistics and performed statistical tests using the bootstrapping resampling method with the 1000 sets of bootstrap weights generated by Statistics Canada. All analyses were performed using the Survey Procedures in version 9.4 of the SAS software (SAS Institute Inc., Cary, NC, USA).

## Results

Respondents were 30 years old on average, slightly more than half were women, and almost all lived in an uncrowded household (Table 1). While men and women did not have significantly different levels of material deprivation, women experienced more social deprivation than men.

**Table 1:** Sociodemographic characteristics of the respondents (*n* = 13,020)

		Participants	Women (54.8%)	Men (45.2%)	***P***
**Age**		**30.6 ± 0.1**	**30.7 ± 0.2**	**30.4 ± 0.2**	**0.16**
**Overcrowding index (persons/room)**	**≤ 1**	**92.2**	**92.2**	**92.2**	**0.24**
	**> 1, < 1.5**	**3.8**	**3.5**	**4.1**	
	**≥ 1.5**	**4.1**	**4.3**	**3.7**	
**Material deprivation (quintile)**	**1st**	**14.1**	**13.4**	**14.9**	**0.50**
	**2nd**	**18.3**	**17.8**	**18.9**	
	**3rd**	**18.5**	**18.7**	**18.2**	
	**4th**	**20.7**	**21.3**	**19.9**	
	**5th**	**28.4**	**28.7**	**28.1**	
**Social deprivation (quintile)**	**1st**	**12.7**	**11.7**	**14.0**	**< 0.001**
	**2nd**	**15.9**	**15.0**	**17.0**	
	**3rd**	**19.1**	**17.6**	**20.9**	
	**4th**	**24.0**	**25.5**	**22.3**	
	**5th**	**28.2**	**30.2**	**25.9**	

Data shown are percentage or mean ± standard error. Differences between men and women were tested with a t-test (age) or chi-square test (proportions)

In terms of intergenerational solidarity, more than one third of the respondents spent time with an elder, and less than one out of ten indicated they would turn to an elder or a grandparent for support in times of need (Table 2). With regards to mental health outcomes, women more often perceived their mental health as fair or poor than men. The proportion of women experiencing mood disorder or anxiety was twice higher than that of men (Table 2). In addition, about one out of 20 respondents had suicidal thoughts in the last 12 months, and this proportion did not significantly differ between men and women. Suicide attempts were about two times more frequent in woman than men.

**Table 2:** Intergenerational solidarity variables and mental health outcomes of respondents

	Participants (%)	Women (%)	Men (%)	***P***
**Intergenerational solidarity**				
**Spent time with an elder**	**39.0**	**40.0**	**37.8**	**0.15**
**Would turn to an elder or grandparent for support**	**8.9**	**9.3**	**8.3**	**0.19**
**Mental health**				
**Perceived mental health (fair or poor)**	**12.2**	**13.9**	**9.9**	**< 0.001**
**Mood disorders**	**14.7**	**19.1**	**9.3**	**< 0.001**
**Anxiety**	**14.6**	**19.0**	**9.1**	**< 0.001**
**Suicidal thoughts (last 12 months)**	**5.3**	**5.7**	**4.8**	**0.23**
**Attempted suicide (last 12 months)**	**1.3**	**1.7**	**0.9**	**0.02**

Data shown are percentage. Differences between men and women were tested with a chi- square test

After adjusting for age and level of material and social deprivation, Indigenous people living off reserve who lacked intergenerational solidarity were more likely to experience mental health issues (Table 3). Men not having spent time with an elder were more likely to have mood disorders (Table 3). Women who mentioned that they would not turn to an elder or grandparent for support in times of need were more likely to report fair or poor perceived mental health and anxiety (Table 3). Moreover, women not having spent time with an elder were more likely to have reported suicidal thoughts in the last 12 months (Table 3). Finally, women who would not turn to an elder or grandparent for support in times of need were more than three times more likely to have attempted suicide.

**Table 3:** Adjusted odds ratios and confidence intervals for the association between (lack of) intergenerational solidarity and mental health outcomes

Mental health conditions	Intergenerational solidarity					
	Did not spend time with an elder			Would not turn to an elder/grandparent for support		
	All	Women	Men	All	Women	Men
Perceived mental health (fair or poor)	1.19 (0.93–1.53)	1.14 (0.83–1.55)	1.36 (0.95–1.93)	1.48 (1.05–2.09)	1.69 (1.06–2.70)	1.23 (0.73–2.06)
Mood disorders	1.13 (0.93–1.39)	1.01 (0.77–1.32)	1.66** (1.18–2.33)	1.24 (0.83–1.84)	1.47 (0.98–2.20)	0.95 (0.43–2.11)
Anxiety	0.98 (0.80–1.19)	0.95 (0.74–1.22)	1.12 (0.80–1.56)	1.32 (0.95–1.83)	1.57* (1.03–2.39)	1.01 (0.61–1.66)
Suicidal thoughts (last 12 months)	1.45* (1.03–2.03)	1.62* (1.02–2.57)	1.25 (0.74–2.11)	1.12 (0.70–1.79)	1.36 (0.77–2.39)	0.84 (0.37–1.91)
Attempted suicide (last 12 months)	1.01 (0.60–1.70)	1.13 (0.61–2.12)	0.80 (0.31–2.08)	1.06 (0.39–2.91)	3.38* (1.06–10.80)	0.29 (0.07–1.19)

Adjusted factor are age and level of material and social deprivation**.** Asterisks represent significant associations: * *p* < 0.05; ** *p* < 0.01

## Discussion

We aimed to verify the association between intergenerational solidarity involving Indigenous elders and mental health outcomes of Indigenous people living off reserve. We found partial association between intergenerational solidarity involving elders and mental health in Indigenous people living off reserve. Men were more likely to have mood disorders if they had not spent time with an elder. Women who would not turn to an elder or a grandparent in times of need were more likely to have anxiety disorders and much more likely to attempt suicide. Women who had not spent time with an elder were more likely to report suicidal thoughts.

As intergenerational solidarity is part of the more general social support from family and friends, our results are in line with those of previous studies that demonstrated the importance of social support for mental health in various populations [29], including Indigenous people [30]. The latter study showed that having higher social support significantly decreased the likelihood of depression and anxiety in women, but not in men. Men and women might experience depression and anxiety differently, or the data collection tools used by Bernards et al. [30] might not have adequately captured social support as experienced by men. Higher perceived social support has been related with fewer symptoms of anxiety and a lower risk of suicidal thoughts and attempts [31, 32]. The higher rate of suicidal attempts in women should be interpreted with caution, as men are less likely to survive after suicidal attempts because they use more drastic means [33]. A meta-analysis showed that having high social connections (subjectively and objectively) was positively associated with good mental health, lower risk of mortality, and better health habits [34]. Specifically, having high social connections was associated with a 50% higher proportion of survival [34], for both men and women. When faced with cultural challenges, social support could be more beneficial to mental health for people with lower socioeconomic conditions and education level [35].

It has been largely shown that positive social connectedness is associated with better psychological wellbeing and mental health [34–36]. In fact, positive interactions with those who are closest such as our intimate friends and family members have significant effect to positively change our mood. At long, these interactions may serve as a buffer against stressors and build resiliency. In opposition, social isolation has adverse consequences on health and mental health. Moreover, loneliness has been associated with depressive symptomatology and suicidal ideation [36]. As a form of social support, indigenous elders contribute to the transmission of values, culture and collective identity, hence fostering community cohesion and development [13, 15]. In the present study, focusing on the role of Indigenous elders in intergenerational solidarity highlighted that their contribution to more general social support improves mental health outcomes of younger generations. Previous studies had shown the key role of intergenerational solidarity within the social support mechanisms that improve mental health [37]. Indeed, Indigenous elders contribute to a sense of belonging to family, community and peers that helps youngers to deal with adversity [13] Moreover, greater social participation increases resilience and sense of community belonging [38] including by indigenous Elders [12]. Resilience allows one to recover good mental health after adverse, stressful or traumatic experiences [39]. A higher sense of community belonging facilitates elder involvement in the teaching of traditional knowledge, values and practices [11–13].

We found a significantly higher rate of social deprivation among Indigenous women, which could explain why they benefited more from intergenerational solidarity than men for most mental health outcomes. Indeed, Belsky et al. [40] demonstrated that intergenerational relations were more beneficial for persons experiencing lower socioeconomic conditions and having less social interactions or support. Considering the benefits of spending time with an elder or turning to an elder or grandparent in times of need, mental health services could be improved by enhancing interactions with elders [22, 23].

Future studies should aim to better understand the differential associations between intergenerational solidarity and mental health outcomes in Indigenous men and women, especially mood disorders for men and anxiety and suicidal thoughts and attempts for women. Future studies using qualitative data (in-depth interviews) should also examine the quality of relationships, beyond merely spending time with elders. Moreover, further research is needed to understand how the association between intergenerational solidarity and mental health outcomes vary according to age, as well as social and material deprivation.

Our study had limitations. First, respondents to the APS could have felt discomfort when prompted by strangers to talk about mental health issues, which may have led to their underreporting due to a social desirability bias [41]. Moreover, for people with a physical or mental disability, the questions were answered by a proxy, possibly not representing the real answers, especially for mental health issues. In addition, proxy variables for intergenerational solidarity might not have allowed us to fully depict the complexity of such associations. Moreover, intergenerational solidarity is one aspect of the larger concepts of social support and social connectedness, which were not considered in the present study specifically interested in the role of elders, of key importance in Indigenous contexts [11–14]. Among the limitations of the study, it should be noted that our analyzes were carried out using pre-existing data, so we did not have access to all the variables that could have been of interest. Even though the association between intergenerational solidarity and mental health issues were adjusted for material and social deprivation, future studies should clarify the importance of broader intergenerational solidarity according to other dimensions of social support and connectedness. Finally, our results only apply to Indigenous people living off reserve. Similar studies are crucially needed for Indigenous people living in reserves and communities, but unfortunately data are currently lacking.

## Conclusions

We found associations between intergenerational solidarity involving elders and mental health outcomes in Indigenous people living off reserve. Our results could guide policies and practices that aim to enhance mental health and wellness in Indigenous populations. With this guidance, Indigenous elders could also be more involved in the development of mental health and wellness programs to foster a holistic approach and cultural safety.

## Data Availability

Output files are available upon request from the corresponding author.

## Abbreviations

APS: Aboriginal Peoples Survey
ICF: International Classification of Functioning, Disability and Health
FNQLHSSC: First Nations of Quebec and Labrador Health and Social Services Commission
NAFC: National Association of Friendship Centres
OR: Odds Ratio
QICSS: Quebec Inter-University Centre for Social Statistics
SAS: Statistical Analysis System
UNICEF: United Nations International Children’s Emergency Fund
WHO: World Health Organization
